# Molecular analysis of human leukocyte antigen class I and class II allele frequencies and haplotype distribution in Pakistani population

**DOI:** 10.4103/0971-6866.73408

**Published:** 2010

**Authors:** T. Moatter, M. Aban, S. Tabassum, U. Shaikh, S. Pervez

**Affiliations:** Department of Pathology & Microbiology, Aga Khan University Hospital, Karachi, Pakistan

**Keywords:** DQB1, DRB1, human leukocyte antigen polymorphism, human leukocyte antigen, *HLA-A*, *HLA-B*, *HLA-C*, Pakistan, population genetics, sequence-specific oligonucleotide analysis

## Abstract

**AIM:**

Distribution of HLA class I and II alleles and haplotype was studied in Pakistani population and compared with the data reported for Caucasoid, Africans, Orientals and Arab populations.

**MATERIALS AND METHODS:**

HLA class I and II polymorphisms in 1000 unrelated Pakistani individuals was studied using sequence-specific primers and polymerase chain reaction and assay.

**RESULTS:**

The most frequent class I alleles observed were A*02, B*35 and CW*07, with frequencies of 19.2, 13.7 and 20%, respectively. Fifteen distinct HLA-DRB1 alleles and eight HLA-DQB1 alleles were recognized. The most frequently observed DRB1 alleles which represented more than 60% of the subjects were DRB1 *03, *07, *11 and *15. The rare DRB1 alleles detected in this study were HLADRB1 *08 and *09, having frequencies of 0.9 and 1.7%, respectively. In addition, at DRB1-DQB1 loci there were 179 different haplotypes and 285 unique genotypes and the most common haplotype was DRB1*15-DQB1*06 which represented 17% of the total DRB1-DQB1 haplotypes. In our population, haplotype A*33-B*58-Cw*03 comprised 2.8% of the total class I haplotypes observed. This haplotype was seen only in the oriental populations and has not been reported in the African or European Caucasoid.

**CONCLUSION:**

Our study showed a close similarity of HLA class I and II alleles with that of European Caucasoid and Orientals. In Pakistani population, two rare loci and three haplotypes were identified, whereas haplotypes characteristic of Caucasians, Africans and Orientals were also found, suggesting an admixture of different races due to migration to and from this region.

## Introduction

The human leukocyte antigen (HLA) system spans a 4-Mb region on the short arm of chromosome 6, band p21.3. It consists of class I, II, and III non-overlapping segments which encode for cell-surface heterodimeric glycoproteins.[1] The genes for these antigens are highly polymorphic and play a central role in the immune response.[2] Allele and haplotype frequencies of the HLA loci differ among various human populations and the studies of polymorphism in HLA system are useful for tracing the evolution of different populations and identification of conserved combination of different alleles.[3,4] Moreover, HLA alleles have been implicated to both disease susceptibility and progression and, in some instances, even resistance to certain diseases because of their crucial role in immune system.[5] Several disease association studies have shown association of different HLA alleles with the same disease in different populations.[6] HLA system also plays a major role in allograft rejection. HLA molecules expressed by transplanted organs are strongly immunogenic, and if not matched with donor HLA, are recognized as non-self and initiate T-cell proliferation and destruction of the transplanted organ, and in association with other cellular and antibody responses, may lead to graft rejection.[7] Therefore, for predicting the probability of finding compatible donors in unrelated bone marrow transplantation, it is valuable to have reliable estimates of HLA allele and haplotype frequencies.

In this study, we carried out molecular analysis of class I *HLA-A, HLA-B, HLA-C* and class II *HLA-DRB1, HLA-DQB1* genes at medium resolution level in a population of Pakistani nationals, using polymerase chain reaction and sequence specific primers (PCR-SSP) technique. The distribution of various allele frequencies and the putative haplotypes are compared with those of different populations.

## Materials and Methods

### 

#### Subjects

In this study, we investigated a sample of 1000 individuals representative of the Pakistani population for the distribution of HLA class I and II alleles. The sample population of 1000 unrelated subjects (men, women) had a mean age 26.7 ± 9.5 years.

#### DNA extraction and HLA-A, B, C, DRB1 and DQB1 typing

Blood samples (5 ml each) of individuals who participated in this study were obtained in tubes containing ethylenediamine tetraacetic acid (EDTA) as anticoagulant. White blood cells were separated on Ficoll/Hypaque and DNA was extracted according to the method of Miller *et al*.[8] HLA alleles were identified using PCR-SSP technique as described by the manufacturer (One Lambda, Canoga Park, CA, USA). The HLA typing method used in this study could discriminate all of the HLA class I and class II alleles officially recognized by the WHO nomenclature committee for Factors of the HLA system.[9] Allele frequencies and haplotype frequencies were calculated using the software Pypop Win32-0.7.0.[10]

## Results

The frequency distribution of HLA-A, -B, -C alleles was obtained by DNA typing of 1000 unrelated healthy Pakistani nationals comprising 340 females and 660 males, representing all the major regions of Pakistan. Sixteen distinct alleles of HLA-A, 28 alleles of HLA-B and 13 different alleles of HLA-Cw were identified [Table 1]. The most frequent class I alleles observed were A*02, B*35 and Cw*07, showing a frequency of 19.2, 13.7 and 20%, respectively. Other most common HLA-A alleles were A*01, *24, *11 and *33, whereas HLA-A *66, *69 and *74 were rare and present in less than 1.5% of the sample population. HLA-B *8, *15, *40, *51 and *52 were detected in a large number of individuals. For HLA-C, six alleles were the most common and were identified in 83% of the subjects. These were Cw *03, *04, *06 *07, *12 and *15. The genotypes of HLA-A, A*02:A*02 and A*02:A*68, showed a significant difference between the observed and expected frequencies (*P* < 0.5). In addition, genotypes of HLA-B, *35:*58, showed statistically significant differences between the observed and expected frequencies (*P* < 0.5).

**Table 1:** Allele frequencies of HLA class I in Pakistani population (n = 1000)

HLA-A allele	Frequency	HLA-B allele	Frequency	HLA-B allele	Frequency	HLA-Cw allele	Frequency
A*01	0.127	B*07	0.049	B*53	0.005	Cw*01	0.058
A*02	0.192	B*08	0.079	B*54	0.001	Cw*02	0.010
A*03	0.078	B*13	0.033	B*55	0.023	Cw*03	0.090
A*11	0.136	B*15	0.073	B*56	0.004	Cw*04	0.143
A*23	0.008	B*18	0.028	B*57	0.036	Cw*05	0.009
A*24	0.121	B*27	0.013	B*58	0.054	Cw*06	0.117
A*26	0.079	B*35	0.137	B*78	0.001	Cw*07	0.202
A*29	0.039	B*37	0.022			Cw*08	0.015
A*30	0.023	B*38	0.010			Cw*12	0.151
A*31	0.032	B*39	0.009			Cw*14	0.033
A*33	0.089	B*40	0.120			Cw*15	0.128
A*34	0.005	B*41	0.012			Cw*16	0.034
A*66	0.005	B*42	0.002			Cw*17	0.010
A*68	0.056	B*44	0.056				
A*69	0.005	B*45	0.005				
A*74	0.005	B*46	0.003				
		B*48	0.004				
		B*49	0.008				
		B*50	0.030				
		B*51	0.104				
		B*52	0.079				

The allele frequencies and distribution of HLA-DRB1 and HLA-DQB1 loci are shown in Table 2. Thirteen distinct HLA-DRB1 alleles and five HLA-DQB1 alleles were recognized. Frequently observed DRB1 alleles were *03, *07, *11 and *15, representing greater than 60% of the samples. The commonest HLA-DRB1 type was *15; its frequency was 21%. DRB1*15 alleles of DR2 family were most frequently found in this study. DRB1*15 constituted 91% of the total DR2 subtypes. Out of 914 HLA-DRB1 genotypes observed, genotype *03:*15 was observed in a smaller proportion of the population than was expected and this difference was statistically significant (*P* < 0.001).

**Table 2:** Frequencies of HLA class II alleles in Pakistani population (n = 1000)

HLA-DRB1 allele	Frequency	HLA-DQB1 allele	Frequency
DRB1*01	0.038	DQB1*02	0.265
DRB1*03	0.168	DQB1*03	0.275
DRB1*04	0.065	DQB1*04	0.010
DRB1*07	0.134	DQB1*05	0.204
DRB1*08	0.009	DQB1*06	0.246
DRB1*09	0.017		
DRB1*10	0.046		
DRB1*11	0.128		
DRB1*12	0.020		
DRB1*13	0.075		
DRB1*14	0.071		
DRB1*15	0.209		
DRB1*16	0.020		

Table 3 shows the estimated frequencies of two-locus haplotypes extended from HLA-DRB1 to HLA-DQB1. Sixty-three different haplotypes and 285 unique genotypes for DRB1-DQB1 were recognized. The most common haplotype was HLA-DRB1*15-DQB1*06; its frequency and relative linkage disequilibrium values were 16.9 and 1.00, respectively. Besides DQB1*06, DRB1*15 was found in association with DQB1*03 and *05. However, for all the different haplotypes for DRB1*15 DQB1 alleles identified, HLA-DRB1*15 was found in strong association with DQB1*06 (82%; *P* < 0.001). HLA-DQB1*02 was recognized to be in association with HLA-DRB1*03 as the second most common haplotype (15.8%) in Pakistani population. No single HLA-DRB1 allele that showed strong association with multiple HLA-DQB1 alleles was identified.

**Table 3:** Estimated frequencies of HLA A:B:C and DR:DQ haplotypes (n = 1000)

Haplotype			Frequency	Haplotype		Frequency
A*02	B*40	C*15	0.052	DRB1*15	DQB1*06	0.169
A*26	B*08	C*07	0.039	DRB1*03	DQB1*02	0.158
A*33	B*58	C*03	0.028	DRB1*11	DQB1*03	0.116
A*11	B*35	C*04	0.024	DRB1*07	DQB1*02	0.100
A*02	B*50	C*06	0.023	DRB1*14	DQB1*05	0.072
A*11	B*52	C*12	0.022	DRB1*13	DQB1*06	0.065
A*24	B*35	C*04	0.021	DRB1*04	DQB1*03	0.063
A*33	B*44	C*07	0.019	DRB1*10	DQB1*05	0.042
A*03	B*35	C*04	0.018	DRB1*07	DQB1*03	0.035
A*02	B*52	C*12	0.017	DRB1*01	DQB1*05	0.032

Out of 2608 haplotypes estimated for HLA-A, -B and C, the frequencies of 10 most common haplotypes which constituted 26% of the total haplotypes are listed in Table 3. The haplotypes A*02, B*40 and Cw*15 were the most frequent in Pakistanis. As shown in Table 3, HLA-A*02 was involved in 9% of the class I haplotypes primarily in association with B*40, B*50, B*52, Cw*15, Cw*06 and Cw*12. Analysis of HLA-B and C associations showed that B*35 was most tightly associated with Cw*04, whereas with HLA-A, strong association of B*40 was identified with HLA-A*02. A total of 2747 unique genotypes and 1757 haplotypes of HLA-A, B and DRB1 were identified in this study [Table 4]. Further analysis of HLA-A, B and DRB1 haplotypes have demonstrated associations and haplotype frequencies of 3.3% for A*26, B*08 and DRB1*03. Other commonly found A:B:DRB1 haplotypes included A*02:B*40:DRB1*15 showing 3% frequency. Estimation of association between HLA-B and HLA-DRB1 alleles shows that the most frequent DRB1 allele *15 was in association with HLA-B*40 and B*52, both of which contributed to more than 10% of the total haplotypes.

**Table 4:** Estimated frequencies of three-locus haplotypes in Pakistani population (n = 1000)

	Haplotype		Frequency
A*26	B*08	DRB1*03	0.033
A*02	B*40	DRB1*15	0.030
A*33	B*58	DRB1*03	0.020
A*02	B*35	DRB1*15	0.015
A*33	B*44	DRB1*07	0.015
A*02	B*50	DRB1*07	0.013
A*01	B*57	DRB1*07	0.011
A*03	B*35	DRB1*04	0.011
A*11	B*40	DRB1*15	0.010
A*24	B*40	DRB1*15	0.010

## Discussion

HLAs are a key player in shaping the ability of the immune system to distinguish between self and non-self. PCR-SSCP based typing has been used to identify many novel alleles at HLA class I and class II loci, making it a necessary tool for studying new alleles with variations in regions previously not known to be polymorphic. HLA system is a useful tool for distinguishing and relating populations. Moreover, the study of HLA frequencies and genetic distances can be used to assess the existence or absence of gene flow among neighboring populations.[11] In this study, multi-locus haplotype analysis was carried out for HLA class I and class II, and the data were compared with those of other populations of the world to evaluate the genetic relations with other populations. Our analysis has shown that for HLA-A, the most frequent allele was A*02. Higher frequency of HLA*02 has been reported in other populations including Caucasians (28%), Russians (26%), South Indians, Kerala Hindu population (25%), and Turks (29%).[12–15] In Pakistani population, HLA-A genotype HLA A*02: A*02 was significantly more common (*P* < 0.05) when compared with other HLA-A genotypes.

The most common HLA B types identified in Pakistan were B*35, *40, *51 and *52. The distribution of HLA-B alleles in our population was comparable to Iranian and Oriental populations, except for HLA B*52 which was proportionally higher in Pakistan (2.6% vs. 8%).[16,17] In Caucasian and Negroid populations, the frequency of B*35, B*40, B*51 and B*52 was lower than that of Pakistani population.[12,16] For HLA-C locus, Cw*07 was found in 20% Pakistani individuals when compared with other populations. Cw*07 was most frequently seen in Russian and Caucasian populations (36% and 32.6%, respectively).[12,13] It was noted that at HLA-C locus, the most common genotype was Cw*07: Cw*12 and rare genotype was Cw*03: Cw*04 (*P* < 0.001).

The analysis of our data shows that the most frequent allele from HLA-DRB1 locus is DRB1*15, with a frequency of 20.5% The frequency of this allele in other populations examined including Iranian (12%), Caucasian (9.3%) and North Africans (10.7%) was relatively lower.[12,18,19] However, similar frequency of HLA-DRB1*15 was noted in North Indian population. In the Far East including Japan and China, the frequencies for DRB1*15 were 2.9 and 4.4%, respectively, compared to 20.5% in the Pakistani population.[20–22] The most frequent DQB1 allele in the Pakistani population was found to be DQB1*02 with a frequency of 39%. This allele existed at a higher frequency in North Indian (32%), Tunisian (32.6%) and African (30.5%) populations.[21–24] In contrast, in the Bulgarian and Chinese populations, its frequency was reported to be 13.5 and 17.8%, respectively.[25,26]

The common two locus haplotype in Pakistani population was DRB1*15-DQB1*06 (17%). This haplotype with a frequency of about 15% was reported in Caucasoids, Africans (26.6%).[23,27] This haplotype is also very common in the Indian population (13.2%), whereas in Iran and Yemeni populations DRB1*15-DQB1*06 was present at a lower level with a frequency of 2.7 and 2.0%, respectively.[28,29] The haplotype DRB1*07-DQB1*02 was identified in the Pakistani population in 12.2% of the normal individuals, this haoplotype was also found at a very high frequency in the Tunisian (19.5%) and Yemeni (22.5%) and Iranian (6.8%) populations, but it was not reported in the Caucasians of the Western Europe.[29,30] The class I haplotype A*02-B*40 identified in the Pakistani population was also reported in the Oriental populations of Hong Kong (1.8%) and Philippines (3.2%).[31] The distribution of HLA haplotypes in the Pakistani population indicates that it has an influence of Caucasians and Oriental populations.

In conclusion, our study has revealed the frequency of HLA class 1 and II alleles and their haplotypes in the Pakistani population. The data presented here may be effectively utilized for analysis of disease association and anthropological studies in the local population, besides its importance in bone marrow transplantation.
